# Genome editing of *Clostridium autoethanogenum* using CRISPR/Cas9

**DOI:** 10.1186/s13068-016-0638-3

**Published:** 2016-10-18

**Authors:** Shilpa Nagaraju, Naomi Kathleen Davies, David Jeffrey Fraser Walker, Michael Köpke, Séan Dennis Simpson

**Affiliations:** 1 LanzaTech Inc, 8045 Lamon Ave, Suite 400, Skokie, IL 60077 USA; 2 Faculty of Medical and Health Sciences, University of Auckland, Auckland, New Zealand; 3 University of Massachusetts, Amherst, MA USA

**Keywords:** Acetogen, CRISPR/Cas9, Gas fermentation, *Clostridium autoethanogenum*, Genome editing, Inducible promoter

## Abstract

**Background:**

Impactful greenhouse gas emissions abatement can now be achieved through gas fermentation using acetogenic microbes for the production of low-carbon fuels and chemicals. However, compared to traditional hosts like *Escherichia coli* or yeast, only basic genetic tools exist for gas-fermenting acetogens. To advance the process, a robust genetic engineering platform for acetogens is essential.

**Results:**

In this study, we report scarless genome editing of an industrially used model acetogen, *Clostridium autoethanogenum*, using the CRISPR/Cas9 system. Initial efforts to retrofit the CRISPR/Cas9 system for *C. autoethanogenum* resulted in poor efficiency likely due to uncontrolled expression of Cas9. To address this, we constructed and screened a small library of tetracycline-inducible promoters that can also be used to fine-tune gene expression. With a new inducible promoter, the efficiency of CRISPR/Cas9-mediated desired gene deletion in *C. autoethanogenum* was improved to over 50 %, making it a viable tool for engineering *C. autoethanogenum*.

**Conclusions:**

Addition of both an inducible promoter library and a scarless genome editing tool is an important expansion to the genetic tool box of industrial *C. autoethanogenum* strain.

**Electronic supplementary material:**

The online version of this article (doi:10.1186/s13068-016-0638-3) contains supplementary material, which is available to authorized users.

## Background

Global greenhouse gas emissions have been rising at an unprecedented rate, with the associated climate instability now being recognized throughout the world by governments as a serious threat to ecosystems, human health, and national economies. To curtail this trend and limit the global temperature rise to 2 °C above pre-industrial levels will require a radical reduction of the use of primary fossil resources for the coming decades [1] and increase the use of low-carbon fuels and chemicals [2] derived from sustainable and waste sources. Gas fermentation offers an opportunity to recycle carbon and harness energy from synthesis gas (syngas) generated from any biomass (such as municipal solid waste, organic industrial waste, or agricultural waste) or industrial off-gases (e.g., from industrial sources like steel mills or processing plants) for the production of transportation fuels and chemical intermediates [3, 4]. The commercialization and at-scale deployment of gas fermentation technology is being actively pursued by several companies with the first commercial units currently under construction [4, 5]. At the heart of the technology are acetogenic bacteria that act as biocatalysts by fixing carbon from gases such as carbon monoxide and/or carbon dioxide in the presence of hydrogen [6]. The principle challenges in commercial exploitation of the vast potential of gas-fermenting acetogens are the relatively basic understanding of acetogens and, in particular, the limited availability of genetic tools and high-throughput genetic engineering platforms [2, 7].

*Clostridium autoethanogenum* is a model acetogen that is being pursued for fuel (ethanol) and chemical (2,3-butanediol) production at commercial scale [4, 5]. However, relatively few genetic tools have been reported for *C. autoethanogenum* [4, 5]. In *C. autoethanogenum*, key insights on the energetics and carbon flux balance have been gained by gene knockout studies using ClosTron, a group II intron-based retrohoming gene disruption tool [8, 9]. However, this intron insertion-based gene inactivation tool has its own limitations as it leaves a huge scar consisting of a fragment of the group II intron along with the antibiotic selection marker. Gene deletions by homologous recombination in *C. autoethanogenum* [10] are achievable but at a very low frequency leading to labor-intensive screening processes and lower efficiencies or by leaving a scar or marker in the genome. A more reliable and stable genetic modification tool that enables scarless genome modifications is preferable.

CRISPR/Cas9 system is an exciting breakthrough in DNA editing technology. Clustered Regularly Interspaced Short Palindromic Repeat, CRISPR, is a bacterial acquired immune system to combat phage infections that has been intelligently adapted for biotechnology purposes [11–13]. CRISPR/Cas9 from *Streptococcus pyogenes* relies on a 20-nucleotide information in its crRNA–tracrRNA chimeric RNA (single-guide RNA, sgRNA) to guide Cas9 endonuclease to the target DNA where it introduces double-stranded breaks (DSB). In most eukaryotes, the DSB are repaired by non-homologous end joining. However, in prokaryotes the repair is by homologous recombination and is mediated by a DNA repair template. CRISPR/Cas9-mediated genome modification has been shown in a diverse array of microbial systems including in a few Clostridia, recently [14–18].

Here we describe the applicability of *Streptococcus pyogenes* type II CRISPR/Cas9 system for genetic modification of *C. autoethanogenum* which already has a type-1B CRISPR [19]. We further show that the adaptation of the heterologous CRISPR/Cas9 system for use in *C. autoethanogenum* required constructing and screening a small library for stronger tetracycline-inducible promoter(s). For the exemplification of the CRISPR/Cas9 system, two genes, namely a NADPH-dependent primary:secondary alcohol dehydrogenase (*adh*; CAETHG_0553) and a 2,3-butanediol dehydrogenase (*2,3*-*bdh*; CAETHG_0385), were chosen. The rationale for targeting these genes is centered on their involvement in ethanol and 2,3-butanediol metabolism [20, 21] and the fact that both genes had been previously inactivated (using ClosTron methodology) without having an impact on growth [10, 22], thus making them predictable targets for genetic tool validation.

## Results and discussion

**Fig. 1:**
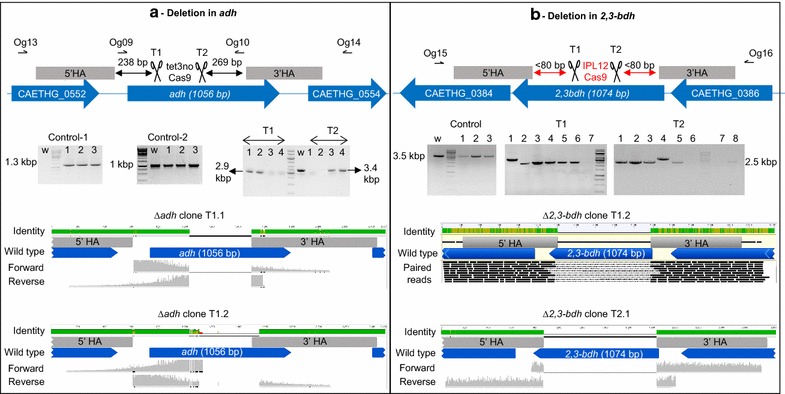
CRISPR/Cas9-based editing of *C. autoethanogenum*. **a**, **b** show design and screening for editing *adh* and *2,3*-*bdh*, respectively. The modifications in the design for editing *2,3*-*bdh* such as the position of the homology arms (5′HA and 3′HA, checkered* gray box*) relative to Cas9 cleavage site (T1 and T2; scissors) and *cas9*-inducible expression from IPL12 are highlighted in *red*. The alignment of sequence from Sanger sequencing of three clones (**a** ∆*adh* clone T1.1, ∆*adh* clone T1.2 and **b** ∆*2,3*-*bdh* clone T2.1) and next-generation sequencing (MiSeq; B, ∆*2,3*-*bdh* clone T1.2) confirms the partial deletion in *adh* and expected deletion in *2,3*-*bdh*

The *cas9* and sgRNA derived from *S. pyogenes* CRISPR/Cas9 system were introduced into *C. autoethanogenum* on two different plasmids, sequentially. Except for in controls, the sgRNA plasmids contained the homology arms (HAs) that served as DNA editing template. While *C. autoethanogenum* maintained sgRNA plasmids, several attempts to introduce a plasmid carrying *cas9* under the control of a native constitutive phosphotransacetylase–acetate kinase promoter [20] were not successful, likely due to toxicity caused by uncontrolled Cas9 protein expression. This was addressed by regulating the expression of *cas9* by a tetracycline-inducible promoter, tet3no [23]. Two sgRNAs with unique binding sites to the target gene (Fig. 1a, b) were individually expressed using a native Wood–Ljungdahl cluster promoter [24].

The *adh* gene was targeted first (Fig. 1a). Following confirmation of the presence of *cas9* by PCR (Fig. 1a, control-1), sgRNA plasmids with (psgRNA-adh-T1_HA and psgRNA-adh-T2_HA) and without HA (psgRNA-adh-T1) were then introduced. The *cas9* expression in colonies transformed with *cas9* and sgRNA plasmids was induced with 32 ng/ml anhydrotetracycline. The induced colonies were then screened for 891 bp deletion within *adh* by PCR using primers flanking the HA (Fig. 1a). In the absence of HA or DNA editing template, no deletion was detected (Fig. 1a, Control-2) and Sanger sequencing of these PCR products did not show insertions/deletions (INDELs). Four colonies were obtained on screening plates with psgRNA-adh-T1_HA and psgRNA-adh-T2_HA. Interestingly, amplicons of ~2.9 kbp instead of a ~2.5 kbp size were detected in two colonies with psgRNA-adh-T1_HA (Fig. 1a, T1, 1 and 2), implying a partial deletion in *adh* rather than the expected ~0.9-kbp deletion. From the remaining two colonies (Fig. 1a, T1, 3 and 4), no fragment was amplified implying a probable integration of the plasmid at the target locus. Sanger sequencing of ~2.5 kbp PCR amplicons confirmed the partial deletion in *adh* (Fig. 1a, ∆*adh* clone T1.1 and 1.2; Additional files 1, 2). The mutants with anticipated length of deletion were not generated. Two of the four colonies from psgRNA-adh-T2_HA amplified fragments corresponding to the wild type (Fig. 1a, T2, 3 and 4), and the remaining two, similar to psgRNA-adh-T1_HA, likely have the plasmid integrated at the targeted locus (Fig. 1a, T2, 1 and 2). This could be likely due to poor recognition of the target site by guide RNA adh-T2.

The partial deletion of *adh* only in the presence of all three components: *cas9*, sgRNA, and DNA editing template indicated the activity of the heterologous CRISPR/Cas9 system in *C. autoethanogenum* and scope for further improvement. To further optimize the CRISPR/Cas9 system for improved performance in *C. autoethanogenum*, two modifications were identified: (1) enhanced control of cas9 expression and (2) positioning one of the HAs close to Cas9 cleavage site.

**Fig. 2:**
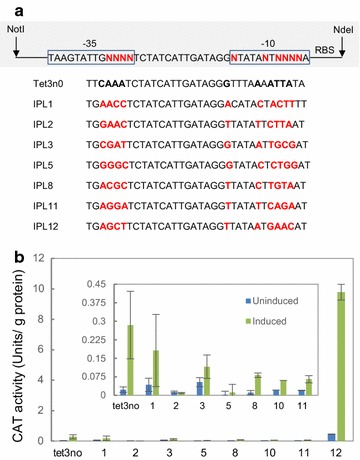
Design and screening of tetracycline-inducible promoter variants with *catP* as a reporter. Shown in **a** are the sequences of the original Tet3n0 promoter and seven synthetic inducible promoters. The randomized non-conserved bases in −35 and −10 promoter elements are highlighted in *red*. Shown in **b** is the activity of *catP* reporter under tet3no and inducible promoters 1–12. The *catP* activity is expressed in Units/g protein. The *inset* shows the *catP* activity of weak promoters on a smaller scale

In order to have an enhanced control over cas9 expression, a set of variants of tetracycline-inducible promoters was constructed based on a method described previously [25] whereby the ten least conserved bases in the −35 and −10 boxes of the rRNA consensus sequences of *C. autoethanogenum* were randomized. For inducible expression, the tet operator (*tet3no*) from the tetracycline-inducible promoter system [26] was inserted in between the randomized −35 and −10 boxes (Fig. 2a). Twelve variants from the inducible promoter library (IPL) were screened with chloramphenicol acetyltransferase gene *catP* as the reporter. Five of these variants could not be grown in liquid media, possibly due to the strength of these promoters. Of the seven remaining promoters, (IPL1 2, 3, 5, 8, 11, and 12), only IPL12 promoter showed significant activity. Even though the non-induced IPL12 promoter showed leaky activity that was higher than the original tet3no promoter, upon induction the activity of IPL12 promoter was approximately ninefold higher than that of tet3no (Fig. 2b). Therefore, the IPL12 promoter was chosen to drive *cas9* expression (Fig. 1b).

The modifications discussed above were tested on a second gene, *2,3*-*bdh*. The expression of *cas9* was driven by IPL12 promoter (IPL12-cas9) and at least one of the HAs was designed to be within 80 bp from Cas9 cleavage site (Fig. 1b) unlike in the previous case where it was at a distance of 250 bp (Fig. 1a) as a measure to avoid potential partial deletion.

Following *cas9* induction, colonies harboring pIPL12-cas9 and one of the two sgRNA plasmids, psgRNA-2,3bdh-T1_HA and psgRNA-2,3bdh-T2_HA, were screened by PCR for deletion in *2,3*-*bdh* using primers flanking the HAs. While a ~3.5-kbp fragment was amplified from wild type and colonies carrying either pIPL12-cas9 or psgRNA-2,3bdh-T1_HA or psgRNA-2,3bdh-T2_HA only (Fig. 1b, control, lanes w, 1, 2, and 3), a deletion product of ~2.5 kbp was observed in colonies (Fig. 1b, T1 lanes 2-6 and T2 lanes 1-3, 5, and 8) carrying *cas9*, sgRNA, and DNA editing template. The desired modification was observed with both sgRNAs targeting *2,3*-*bdh*. This ~1-kbp deletion within *2,3*-*bdh* was further confirmed by sequencing the PCR products from sgRNA T1, clone 2 (Fig. 1b, ∆*2,3*-*bdh* clone T1.2; Additional file 3), and sgRNA T2 clone 1 (Fig. 1b, ∆*2,3*-*bdh* clone T2.1; Additional files 1, 4). The plasmids from positive clones were cured while maintaining the gene deletion by passaging the strains twice on non-selective agar plates. With five out of six and four out of eight colonies harboring *cas9*, DNA editing template, and either sgRNA T1 or T2 having desired deletion in *2,3*-*bdh*, the efficiency of the re-designed system to introduce desired deletion was >50 % compared to the previous attempt.

## Conclusions

In conclusion, the data reported herein demonstrate the workability of the CRISPR/Cas9 tool in *C. autoethanogenum*. In order to efficiently work in *C. autoethanogenum,* the CRISPR/Cas9 system requires the controlled expression of *cas9* and the constitutive expression of sgRNA in the presence of DNA editing template. The new IPL12 tetracycline-inducible promoter significantly increased the efficiency of Cas9-mediated genome editing. Even with screening a relatively small library, promoters with a wide range of expression strengths ranging between the original tet3no to the strong IPL12 promoter were obtained. The developed promoter library has the added potential of expanding the prospective applications of this approach in the metabolic engineering of acetogens. With our modifications, we achieved >50 % efficiency in gene deletion, which is comparable to the efficiencies reported in other Clostridia. The efficiency of CRISPR/Cas9 system adapted for *Clostridium beijerinckii* is unclear [15, 27]. However, Li et al. reported editing efficiencies of up to 100 % in *C. beijerinckii* and *Clostridium acetobutylicum* with the use of nickase variant of *cas9* [18]. In *Clostridium cellulolyticum*, gene deletions were only possible with an engineered nickase variant of *cas9* as the wild-type *cas9* could not be introduced in *C. cellulolyticum* [14]. The use of an inducible promoter to control the expression of wild-type *cas9* would have probably been sufficient to overcome the problem of expressing wild-type *cas9* in *C. cellulolyticum.* Likewise, combining an engineered nickase with the inducible promoter may add additional benefit. In *Clostridium ljungdahlii*, gene deletions with 50–100 % efficiency have been reported with a single-plasmid system comprising both *cas9* and guide RNA expression cassettes with Pthl and ParaE constitutive promoters driving the expression of *cas9* and guide RNA, respectively [17]. The expression of *cas9* from a constitutive promoter could have been likely possible due to the absence of a CRISPR system in *C. ljungdahlii* [19]. The expression of guide RNA from *C. acetobutylicum’s* ParaE promoter, similar to that in *C. ljungdahlii*, and the use of nickase-only Cas9 variant may further improve the efficiency of the CRISPR/Cas9-based genome editing in *C. autoethanogenum*. The above CRISPR/Cas9-based genome editing strategy can be further adapted for gene insertions and to create multiple gene knockouts [11, 12].

## Methods

### Strain and cultivation

A derivative strain of *C. autoethanogenum* type strain DSM10061 [28] was obtained from Deutsche Sammlung von Mikroorganismen und Zellkulturen (DSMZ), Germany, and grown under strict anaerobic condition as described earlier [21].

### Construction of CRISPR/Cas9 plasmids

The Type II CRISPR/Cas9 system from *Streptococcus pyogenes* was used in this study [13]. The *cas9* gene was codon adapted to *C. autoethanogenum* using GenScript’s algorithm and *C. autoethanogenum* codon frequency table. The codon-adapted *cas9* gene (GenBank: KU867916) was synthesized by GenScript. The gene was cloned into vectors pLZtet3no [23] and pIPL12 (described below) between *Nde*I and *Nhe*I restriction endonuclease sites. The resulting vectors are referred as ptet3no-cas9 and pIPL12-cas9. The expression of *cas9* is under the control of an anhydrotetracycline-inducible promoter, tet3no [23] in ptet3no-cas9, and a variant of tet3no promoter in pIPL12-cas9 (discussed below).

Two genes, *adh* and *2,3*-*bdh*, were targeted by CRISPR/Cas9 system. Two sgRNAs, adh-T1 (5′-TAATTGGTATAGGAGCTGT-3′) and adh-T2 (5′-CAATCGCATCATAAGGACC-3′) for *adh* and 2,3bdh-T1 (5′-GAAGGAAATTCATGTCTGT-3′) and 2,3bdh-T2 (5′-TGAAATGGTGTGGTATATG-3′) for *2,3*-*bdh*, were designed and synthesized by GenCRISPR, GenScript’s CRISPR services platform. The specificity of all sgRNAs was tested against *C. autoethanogenum* genome sequence (GenBank accession number CP006763) [19] and two with unique binding site to the target gene preferably to the start and center of genes (Fig. 2a, b) were chosen for further study. The sgRNA was introduced into plasmid pMTL83157 [24] between *Nde*I and *Nhe*I. The ~1 kbp 5′ and 3′ homology arms (HAs) of *adh* (CP006763; position 609,136–610,050 and 610,518–611,451, respectively) and *2,3*-*bdh* (CP006763; position 412,243–412,231 and 414,199–415,144, respectively) were PCR amplified from *C. autoethanogenum* using oligonucleotides listed in Table 2. The HAs were cloned into sgRNA plasmids at *Fse*I restriction site for *adh* and *Pme*I restriction site for *2,3*-*bdh*. The resulting vectors are referred as psgRNA-adh-T1_HA, psgRNA-adh-T2_HA, psgRNA-2,3bdh-T1_HA, and psgRNA-2,3bdh-T2_HA, respectively.

### Construction of variants of inducible promoter

To construct a variant set of inducible promoters, a long oligonucleotide was synthesized by Integrated DNA Technologies (IDT) containing the randomized sequences between the −35 and the −10 boxes in the rRNA consensus sequences (Fig. 1a), a ribosomal binding site (RBS), and the start codon of the chloramphenicol acetyltransferase (*catP*) gene (GenBank EF525477.1). This was annealed at its 3′ end to the start codon of the *catP* gene, and using a reverse oligonucleotide Og17 (annealing to the 3′ end of the *catP*), a large (~864 bp) fragment incorporating these elements was amplified. This fragment was cloned using *Cla*I and *Nhe*I into the pLZtet3no [23] plasmid. The *catP* gene cloned downstream of tet3no-inducible promoter between *Nde*I and *Nhe*I restriction sites in pLZtet3no [23, 29] was used as a reference.

### Strain construction

All vectors were introduced into *C. autoethanogenum* via conjugation as described previously [9]. Strains of *C. autoethanogenum* carrying ptet3no-cas9, pIPL12-cas9, psgRNA-2,3bdh-T1_HA, or psgRNA-2,3bdh-T2_HA were first constructed. Following the growth of colonies carrying these plasmids on agar plates containing 5 µg/ml clarithromycin or 7.5 µg/ml thiamphenicol and 10 µg/ml trimethoprim (to counter-select *E. coli* conjugation donor strain), they were screened to confirm the presence of *cas9* by PCR using oligonucleotides Og05 and Og06 (Fig. 1a, control-1). A *C. autoethanogenum* strain bearing the tet3no-cas9 was re-transformed with plasmids psgRNA-adh-T1_HA or psgRNA-adh-T2_HA. Similarly, a *C. autoethanogenum* strain carrying pIPL12-cas9 was re-transformed with plasmids psgRNA-2,3bdh-T1_HA or sgRNA-2,3bdh-T2_HA. Following outgrowth on selective agar plates consisting of 7.5 µg/ml thiamphenicol (to propagate sgRNA plasmids), 5 µg/ml clarithromycin, and 10 µg/ml trimethoprim, colonies were streaked on plates also containing 32 ng/ml anhydrotetracycline (Sigma; 37,919) to induce the expression of *cas9*. The resulting colonies were screened for modification in *adh* or *2,3*-*bdh* locus using oligonucleotides Og13/Og14 and Og15/Og16, respectively. Using a similar protocol, transconjugants carrying ptet3no-cas9 and sgRNA-adh-T1 without HA were constructed. The *adh* locus in transconjugants carrying ptet3no-cas9 and sgRNA-adh-T1 without HAs or DNA editing template was screened using primers Og09/Og10 (Fig. 1a, control-2). All conjugation experiments with plasmids carrying sgRNA and HA were performed in at least duplicate.

The lists of all plasmids and oligonucleotides with sequences used in this work are listed in Tables 1 and 2, respectively.

**Table 1:** List of plasmids used in this study

Plasmid	Features	References
pLZtet3no	Original tet3no promoter derived from *Clostridium* base vector pMTL82251 with pBP1 replicon, *ermB* marker and *catP* as a reporter gene	[23]
pTet3no-cas9	Cas9 between *Nde*I and *Nhe*I sites in pLZtet3no	This study
pIPL1	Tet3no promoter in pLZtet3no is replaced with IPL1 promoter	This study
pIPL2	Tet3no promoter in pLZtet3no is replaced with IPL2 promoter	This study
pIPL3	Tet3no promoter in pLZtet3no is replaced with IPL3 promoter	This study
pIPL5	Tet3no promoter in pLZtet3no is replaced with IPL5 promoter	This study
pIPL8	Tet3no promoter in pLZtet3no is replaced with IPL8 promoter	This study
pIPL11	Tet3no promoter in pLZtet3no is replaced with IPL11 promoter	This study
pIPL12	Tet3no promoter in pLZtet3no is replaced with IPL12 promoter	This study
pIPL12-cas9	Cas9 between *Nde*I and *Nhe*I sites in pIPL12	This study
pMTL83157	pMTL83151 vector P_WL_ promoter from *C. autoethanogenum*	[24]
pgRNA-adh-T1	pMTL83157 with gRNA T1 targeting *adh* gene	This study
pgRNA-adh-T2	pMTL83157 with gRNA T2 targeting *adh* gene	This study
pgRNA-2,3bdh-T1	pMTL83157 with gRNA T1 targeting *2,3bdh* gene	This study
pgRNA-2,3bdh-T2	pMTL83157 with gRNA T2 targeting *2,3bdh* gene	This study
pgRNA-adh-T1_HA	pMTL83157 with gRNA T1 targeting *adh* gene with homology arms	This study
pgRNA-adh-T2_HA	pMTL83157 with gRNA T2 targeting *adh* gene with homology arms	This study
pgRNA-2,3bdh-T1_HA	pMTL83157 with gRNA T1 targeting *2,3bdh* gene with homology arms	This study
pgRNA-2,3bdh-T2_HA	pMTL83157 with gRNA T2 targeting *2,3bdh* gene with homology arms	This study

The table also includes the main features of plasmids

**Table 2:** List of oligonucleotides used in this study

Oligonucleotide	Sequence (5′ → 3′)	Purpose
Og01	GATTATAAGCGGCCGGCCATAAACTATTTTTTAAAGATAAAAGCT	PCR amplification of 5′-HA of adh
Og02	TACGCCGCCAGGTTTAACCAAAACCAGCTTGGACTTCTAAAGA	PCR amplification of 5′-HA of adh
Og03	TCTTTAGAAGTCCAAGCTGGTTTTGGTTAAACCTGGCGGCGTA	PCR amplification of 3′-HA of adh
Og04	CAACTTGCCCACTGGCCGGCCTGACTATTTCACTATGAGTAAATGGT	PCR amplification of 3′-HA of adh
Og05	GAATGTGTTTAAACTCTCTGAAACTAGCAAATTTGG	PCR amplification of 5′-HA of 2,3bdh
Og06	GAGATAATTATGAAAGCTGTATTGTGGTTGTAAAAGAAGGATTTGAAACAC	PCR amplification of 5′-HA of 2,3bdh
Og07	GTGTTTCAAATCCTTCTTTTACAACCACAATACAGCTTTCATAATTATCTC	PCR amplification of 3′-HA of 2,3bdh
Og08	AAAGGAGTTTAAACGAAAGTGAGCTTTTTGGTTATGAAAA	PCR amplification of 3′-HA of 2,3bdh
Og09	TATTAACCTTATAAAGTCCTACCCC	For screening
Og10	TAATCCTCCTCTTATAGTTTTGTGA	For screening
Og11	CAAAAGCTATACTTAGTGCTAGATT	For screening
Og12	TCATTTCTCTATCTTCAAAAAGTGT	For screening
Og13	AGCTGTAGATAACAATGGGATCAT	For screening
Og14	GTGAGATATAATGAGAAACCTGATCC	For screening
Og15	AATGGCAGGGCAGATAATTGTAATG	For screening
Og16	AAGGCATTCTGAGCCAGTTCTTTTA	For screening
Og17	TAACGTCCTTAACTATTTATCAATTCGATCGACTAT	To construct variants of ptet3no

The table also includes the purpose of each oligonucleotide

### Chloramphenicol acetyltransferase (CAT) assay

*Clostridium autoethanogenum* strains containing plasmids with the synthetic inducible promoter variants were grown on PETC-MES media supplemented with clarithromycin (5 µg/ml) until the cell density reached OD_600_ of 1. The cells were then sub-cultured to an OD_600_ of 0.1, and grown until an OD_600_ of 0.5 was reached. At this stage, the culture was split into 2 volumes, with one being induced with 31.6 ng/µl of anhydrotetracycline and the other left non-induced. The cultures were grown under these conditions for 6 h, and 2 ml of culture was pelleted and resuspended in 1 ml phosphate buffered saline buffer. Cells were sonicated at 20 mA, for 30 s on and 30 s off for 6 cycles. Following sonication, the debris was pelleted, and the supernatant was used for CAT assays as described earlier [30].

### Sanger sequencing and data analysis

The cleaned PCR products were Sanger sequenced by QuintaraBio (http://www.quintarabio.com/services). The resulting ABI chromatograms were processed with Geneious version 9.0.5 software (http://www.geneious.com, [31]) that automatically calls bases with consensus threshold at 85 %. The sequences were then aligned to reference which was either *adh* (CP006763; 608,975–611,467) or *2,3*-*bdh* (CP006763; 411,994–415,316) locus.

### MiSeq and data analysis

The cleaned PCR product of ∆2,3-bdh clone T1.2 was subjected to MiSeq sequencing in-house. The Nextera DNA Library Preparation Kit from Illumina was used to prepare the library as per the protocol recommended by the supplier. The library was sequenced on MiSeq instrument to get 2× coverage with reads of 150 bp. The 2 × 2879 reads were paired and the resulting paired reads assembly was mapped to the reference *2,3*-*bdh* locus (CP006763; 411,994–415,316) using built-in Read Mapper in Geneious version 9.0.5 software. The consensus sequence was generated using highest quality threshold that uses chromatogram quality to call the best base.

## Additional files

**Additional file 1.** Sanger sequences; this file contains Sanger sequence of *adh* and *2,3*-*bdh* loci from ∆adh clone T1.1, ∆adh clone T1.2 and ∆2,3-bdh clone T2.1.

**Additional file 2.** Chromatograms for *adh*; this file contains ABI chromatograms of Sanger sequence of *adh* locus from ∆adh clone T1.1 and ∆adh clone T1.2.

**Additional file 3.** MiSeq read assembly; this file contains MiSeq sequence reads from ∆2,3-bdh clone T1.2 mapped to *2,3*-*bdh* locus using default parameters in Geneious software.

**Additional file 4.** Chromatograms for *2,3*-*bdh*; this file contains ABI chromatograms of Sanger sequence of *2,3*-*bdh* locus from ∆2,3-bdh clone T2.1.

## Abbreviations

CRISPR: Clustered Regularly Interspaced Short palindromic Repeat
sgRNA: single-guide RNA
DSB: Double Stranded Break
*adh*/Adh: NADPH-dependent primary:secondary alcohol dehydrogenase
*2,3*-*bdh/*2,3-Bdh: 2,3-butanediol dehydrogenase
HA: Homology Arm
INDELs: Insertions/Deletions
bp: base pairs
kbp: kilo base pairs
IPL: Inducible Promoter Library
