# Supplementary material for: Genome editing of Clostridium autoethanogenum using CRISPR/Cas9
Source: Biotechnol Biofuels. 2016 Oct 18;9:219. doi: 10.1186/s13068-016-0638-3 (PMC5069954; doi:10.1186/s13068-016-0638-3)
Supplement: Supplementary file 1 — Additional file 1. Sanger sequences; this file contains Sanger sequence of adh and 2,3-bdh loci from ∆adh clone T1.1, ∆adh clone T1.2 and ∆2,3-bdh clone T2.1. [file 13068_2016_638_MOESM1_ESM.docx]

**>Sanger sequence of ∆adh clone T1.1**

TTTAAAGATAAAAGCTTTAAACTTAAAAAACCTATCCAATAGTAAGACCTGATATATATCAAAAGCTCTTAAATTATAATTGGCCCCGGAAATGTAAGAGAATTGGAAAATTGTATTGAAAATATCGTAAATATGAATGGAAATACATCTTTCAACTTCGAAAATAGTATTTCAGTAAATACGCAAACTAGTCCTTGTACTACAAAATTTAAATATGATATGTATTCATTAAAAGAGTTGGAAAAAGAAGCAATAACAAATTGTATGAGTAATTGCAATGGTAACATTGCAAAAGCTTCTAAAATTCTGGGAATAAATAGAAGTACTTTGTATACAAAAATAAAAAAATATCAAATTAATTTTTCTTAAAGTGTATGTAAACACAACTTTGTTGTAAAAAGCAACATTATTTTCTTAAAAAATGTTGCTTTTTACAGCATTTTTCAATTATATATATTAACCTTATAAAGTCCTACCCCCCTAAATTCAACCTTTTCATGATAAAAAACATACTGGCACAACATTTGCTTATATATTTAAATAGATATTTAAAAATACCATTTTATTTACTAAAATTTTTATATTTAATTTATTAGGAGGTTCTATTATGAAAGGTTTTGCAATGTTAGGTATTAACAAATTAGGATGGATTGAAAAGAAAAACCCAGTGCCAGGTCCTTATGATGCGATTGTACATCCTCTAGCTGTATCCCCATGTACATCAGATATACATACGGTTTTTGAAGGAGCACTTGGTAATAGGGAAAATATGATTTTAGGCCATGAAGCTGTAGGTGAAATAGCCGAAGTTGGCAGCGAAGTTAAAGATTTTAAAGTTGGCGATAGAGTTATCGTACCATGCACAACACCTGACTGGAGATCTTTAGAAGTCCAAGCTGGTTTTGGTTAAACCTGGCGGCGTAATTTCTAACATCAACTACCATGGAAGCGGTGATACTTTACCAATACCTCGTGTTCAATGGGGCTGCGGCATGGCTCACAAAACTATAAGAGGAGGATTATGCCCCGGCGGACGTCTTAGAATGGAAATGCTAAGAGATCTTGTTCTATATAAACGTGTTGATTTGAGTAAACTTGTTACTCATGTATTTGATGGTGCAGAAAATATTGAAAAGGCCCTTTTGCTTATGAAAAATAAGCCAAAAGATTTAATTAAATCAGTAGTTACATTCTAAAAATTCATATAAAAAAACTGTCGCATTAAAAAAATGTGACAGTTTTTACTTAAAATATTGGACAAAAGACTTCCTTTCTTATAGATGCAAAAAATAATCAATCCGATTCATAATTATAAACAGAGTTCTTGGCATCAGGTTGAGTTTTGACTCCACCTGATGCTTAGAAATCGTTATCCAGGGCGCGTAACAGTACTTATTCCCCAACTTTGATAGAAAGATTTGGGGTGTTAGCAATGGTAGCGATCGGGATAAACTGATAAATATTTCGTATTTTTAATTGGCGAACTT

**>Sanger sequence of ∆adh clone T1.2**

TCTTAAGTTCGCAATTAAAAATACGAATATTTATCAGTTTATCCGATCGCTACCATTGCTAACACCCAAATCTTCTATCAAAGTTGGGGAATAAGTACTGTTACGCGCCCGGATAACGATTTCTAAGCATCAGGTGGAGTCAAAACTCAACGTGATGCCAAGAACTCTGTTTATAATTATGAATCGGATTGATTATTTTTTGCATCTATAAGAAAGGAAGTCTTTTGTCCAATATTTTAAGTAAAAACTGTCACATTTTTTTAATGCGACAGTTTTTTTATATGAATTTTTAGAATGTAACTACTGATTTAATTAAATCTTTTGGCTTATTTTTCATAAGCAAAAGGGCCTTTTCAATATTTTCTGCACCATCAAATACATGAGTAACAAGTTTACTCAAATCAACACGTTTATATAGAACAAGATCTCTTAGCATTTCCATTCTAAGACGTCCGCCGGGGCATAATCCTCCTCTTATAGTTTTGTGAGCCATGCCGCAGCCCCATTGAACACGAGGTATTGGTAAAGTATCACCGCTTCCATGGTAGTTGATGTTAGAAATTACGCCGCCAGGTTTAACCAAAACCAGCTTGGACTTCTAAAGATCTCCAGTCAGGTGTTGTGCATGGTACGATAACTCTATCGCCAACTTTAAAATCTTTAACTTCGCTGCCAACTTCGGCTATTTCACCTACAGCTTCATGGCCTAAAATCATATTTTCCCTATTACCAAGTGCTCCTTCAAAAACCGTATGTATATCTGATGTACATGGGGATACAGCTAGAGGATGTACAATCGCATCATAAGGACCTGGCACTGGGTTTTTCTTTTCAATCCATCCTAATTTGTTAATACCTAACATTGCAAAACCTTTCATAATAGAACCTCCTAATAAATTAAATATAAAAATTTTAGTAAATAAAATGGTATTTTTAAATATCTATTTAAATATATAAGCAAATGTTGTGCCAGTATGTTTTTTATCATGAAAAGGTTGAATTTAGGGGGGTAGGACTTTATAAGGTTAATATATATAATTGAAAAATGCTGTAAAAAGCAACATTTTTTAAGAAAATAATGTTGCTTTTTACAACAAAGTTGTGTTTACATACACTTTAAGAAAAATTAATTTGATATTTTTTTATTTTTGTATACAAAGTACTTCTATTTATTCCCAGAATTTTAGAAGCTTTTGCAATGTTACCATTGCAATTACTCATACAATTTGTTATTGCTTCTTTTTCCAACTCTTTTAATGAATACATATCATATTTAAATTTTGTAGTACAAGGACTAGTTTGCGTATTTACTGAAATACTATTTTCGAAGTTGAAAGATGTATTTCCATTCATATTTACGATATTTTCAATACAATTTTCCAATTCTCTTACATTTCCGGGCCAATTATAATTTAAGAGCTTTTGATATATATCACGTCTTACTATTGGAATAGTTTTTTAAGTTTAAAAGCTTTTATCTTTAAAAAATAGTTTATCAGT

**>Sanger sequence of ∆2,3-bdh clone T2.1**

CTGATATTATCATACCACAGTAATTGTACTTCCTCTCTTCCCTTTATTTAAGAAGAAATGGAGCTGCAGCAATCATTCCTTCGGAAATTACTCTCTTCACCAGGAACTACTAAAAGTAATAAGTTTCCCTTAAAGTTTCTCTCTTTACTGAATTCTCTTAAAAGTTCCATATTGAGGGCCACTCCAAACTTCATGTCTGCAGTTCCCCTTCCAAATATCCAATCTCCTGATTTAAAATCCTCCATAGCATCTTCATCTAAATTCAATTCTGAGATTCTTTTAGTACATTCATCTACATCAAAAGCCATAGATTTTAAATGTCCAAATTCTTCTACTCCAACTACATCCAAATGACCTGAAAGTATAAACGAATTTGGTGAATTTTCATTTCCATTTACTACTGCCCATACAAAGCTTCTTCCTAAAGGATCATCTTCAATTTGCTCTATTCCTAGATTTTCAGGATTGTCCTTAAAATAAGGTATTTCCAAAATTTTTTCATATATTTTTTCTGCTGCCGCACACTCTTCTTTTGTACCAGATATGCTTGGCACAGATATTAGTTCCCTCATTGTATCGTATACATTTTTCTTAAAGCTCATTTTTGGCCTCCTCAATATAACTGAAAACTTTCAATTACATCTAAGTATACCATTATTTTTATATAAATGTTAATTTACAACAAAAAAAATGAATTCAACCACATAATTTGCTGAATTCATTTTTTAATCAATTCTATGAGTACTTTTTACAATAAGGATTTGTCAGGAGTTACAATTATTTTTACATGTTTTTTCTTTTCAGGTCCTGTAAGTGTTTCAAATCCTTCTTTTACAACCACAATACAGCTTTCATAATTATCTCTCCTTTTTTATAATAGTATGGCAATATTAAAATTTACATATACCTGTTAATATATTCACTATATTTTATAAGCAAAAGCCATACCAAAAACAAGAAACCTGTTAAAAAGACATTTAAAATCACTATGTGCCATTATGACACATACTTAGTGTCCTATTTTGAGAATCTTACTCTTTTCTGGTCATTTTGACACACTTTTTATTCCATACTTTTTCATTTTTCTATACATGGTAGATCTACTGATATTTAAAGATTTTGCAGCTTTTAATATATTTCCCTTACACTTTTTAAGTGCATTTTCAATGTTTTCTTTTTCTAAAACATCCATTGGAAGCACACTAATATTTACTGGATCAGAGGAGACATTTTTTTCATAAACTTCTTTTTCTAAATAATCCAAAGGGGCCATCTTATCCTCACTTAAATAGTAATCCCTCTCTATTACATTTCTAAGTTCTCTAACATTTCCAGGCCAATCATAAGCTTTAATCTTTTCTATGTATGCTTTTTCCACTACTTTCTTCTTACACAAACTTTTAGAATTCAATTCTTCCATAAAATATTTAATTAAAAGCTCTATATCTTCTTTTCTTTCCCTAAGTGGGACAGTTTTTATATTCATCACACTCAATCTATAATAAAGGTCACTTCTGAAATTTTTCTTTTTAATTTCATCCTTGAGCACCCTGTTTGTAGCTCCTATTATCCTTACATTTAGCTGTTTTTCATAAGTTCCTCCAACTCTTGTAATTTTATTATTATCAAGAACCCTTAAAAGCTTTGACTGTATATCAAGAGGAAGCTCTCCTACTTCATCCAAAAAAATAGTTCCTCCATCTGCTAATTCAAACTTTCCAGGCTTTCCTT
